# Epidemic malaria dynamics in Ethiopia: the role of self-limiting, poverty, HIV, climate change and human population growth

**DOI:** 10.1186/s12936-022-04161-2

**Published:** 2022-04-27

**Authors:** Felipe Augusto Maurin Krsulovic, Timothy Peter Moulton, Mauricio Lima, Fabian Jaksic

**Affiliations:** 1 Departamento de Ecología, Facultad de Ciencias Biológicas, Pontifícia Universidad Católica de Chile, Santiago, Chile; 2 Center of Applied Ecology and Sustainability (CAPES), Santiago, Chile; 3 Departamento de Ecologia, Faculdade de Ciéncias Biológicas, Universidade do Estado do Rio de Janeiro, Rio de Janeiro, Brasil

**Keywords:** Malaria dynamics, Climate, HIV, Poverty, Social instability, Population size

## Abstract

**Background:**

During the last two decades, researchers have suggested that the changes of malaria cases in African highlands were driven by climate change. Recently, a study claimed that the malaria cases (*Plasmodium falciparum*) in Oromia (Ethiopia) were related to minimum temperature. Critics highlighted that other variables could be involved in the dynamics of the malaria. The literature mentions that beyond climate change, trends in malaria cases could be involved with HIV, human population size, poverty, investments in health control programmes, among others.

**Methods:**

Population ecologists have developed a simple framework, which helps to explore the contributions of endogenous (density-dependent) and exogenous processes on population dynamics. Both processes may operate to determine the dynamic behaviour of a particular population through time. Briefly, density-dependent (endogenous process) occurs when the per capita population growth rate (R) is determined by the previous population size. An exogenous process occurs when some variable affects another but is not affected by the changes it causes. This study explores the dynamics of malaria cases (*Plasmodium falciparum* and *Plasmodium vivax*) in Oromia region in Ethiopia and explores the interaction between minimum temperature, HIV, poverty, human population size and social instability.

**Results:**

The results support that malaria dynamics showed signs of a negative endogenous process between R and malaria infectious class, and a weak evidence to support the climate change hypothesis.

**Conclusion:**

Poverty, HIV, population size could interact to force malaria models parameters explaining the dynamics malaria observed at Ethiopia from 1985 to 2007.

## Background

During the last two decades, researchers have suggested that the increase of the malaria burden was driven by climate change [1–8]. These studies lead to extensive debates about the importance of climate in the malaria burden in these locations [9–19]. Despite the evident relationship between malaria and climate, principally between seasons, other variables could be involved in the increase of the malaria burden. Globally, the literature suggests that the positive trends in malaria could also be affected by the spread of the Human Immunodeficiency Virus (HIV) [20–24], poverty level [25–27], health campaigns [27–30] and human population size [31, 32]. Alonso et al. [6] have suggested that although temperature was the main driver of malaria dynamics in Kericho tea plantations, temperature could interact with rural population size and HIV prevalence. A recent study [19] found that the HIV incidence together with rural population size could also influenced malaria dynamics in Kericho district in Kenya suggesting that climate had a negligeable effect on malaria dynamics.

**Fig. 1:**
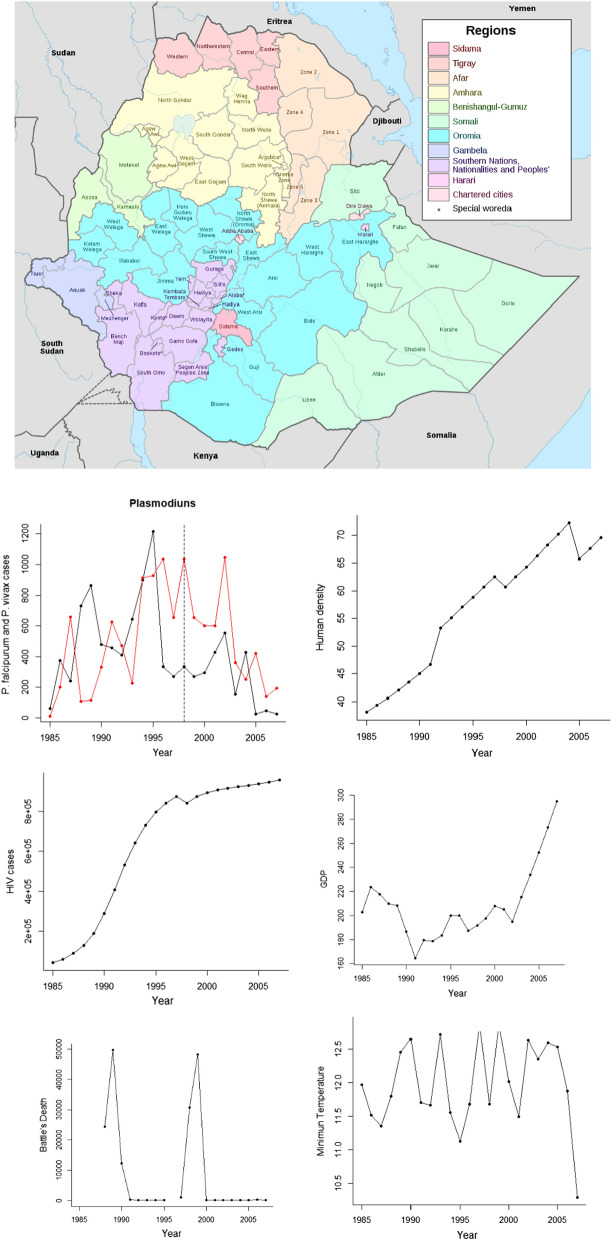
Location of Oromia zone (in blue inside Ethiopia). The site was the Debre Zeit are near of Addis Abada, with meteorological stations ranging from 1600 to 2500 m above sea level. Annual data for *Plasmodiums* annual cases (*P. vivax* in red, vertical dash line separates two chronological periods claimed in the study 2020), HIV new cases, Per capita GDP, Human density, annual Minimum temperature and death related to civil conflicts as a proxy of social instability. Map was taken from (Sarah Vaughan, Archived August 13, 2011), at the Wayback Machine (University of Edinburgh: Ph.D. Thesis, 2003), p. 240 n. 259)

In anoher recent study [16], authors have claimed that the dynamics of the monthly numbers of malaria (*Plasmodium falciparum*) cases were closely related to changes in minimum temperature levels in the region Oromia in Ethiopia, which increased until 1998, them, stabilized and declined from 2000 to 2005 (Fig. 1). In 2005, Ethiopia joined in a global campaign to eradicate malaria [16]. Hence, the authors suggested that the reduction of both malaria cases and minimum temperature (2000–2005) were linked and climate could act synergically with malaria interventions from 2005 on. In resume, climate was the main driver of malaria dynamics in Ethiopia prior the introduction of control programmes. In this study, the data was re-analysed from 1985 to 2007 from the study [16] testing the temperature effects and other variables using inter-annual (annual averages of monthly minimum temperatures and malaria cases) data for malaria dynamics in Ethiopia.

After three decades of decline at global level, around 1980, malaria started to rise and once again became a major health problem, principally in Africa. During the same period (the 1980s decade), HIV started to spread globally [19, 25, 26]. HIV may increase malaria contagion, accelerate progression rates from latent to infectious stage and may delay the recovery of infected individuals, which increases the period in which an infected individual may transmit malaria. In addition, individuals only acquire malaria immunological defenses following several episodes. By affecting the immunological system, HIV removes the acquired defenses (i.e. herd immunity) increasing the susceptible pool (the population at risk of infection) [33, 34].

The increase of human population density (rural and urban) may affect malaria dynamics through reducing the distance between infected and susceptible individuals. Also, the increase of population size expands agriculture and urban frontiers over natural rural areas [19, 35, 36]. This expansion requires deforestation. Deforestation changes the *Anopheles* spp. aquatic food web structure, usually freeing larvae from predators [35]. Deforestation may also increase the number of mosquito breading sites and accelerates the mosquito life cycle by means of an increase in local temperature, resulting in high mosquito population biomass and malaria transmission in rural areas and cities [31, 32, 35, 36], like in the Oromia district in Ethiopia.

Ethiopia is a low-income country composed of ethno-regions (districts). Poverty and political instability, including civil war (between districts) could also affect diseases dynamics and/or epidemic episodes. Malaria, HIV and many others diseases are related to poverty. Difficult access, low-quality health system and inefficient control programmes results in high diseases’ burdens. Social and political instabilities (like civil conflicts) may also impact diseases by concentrating refugees in low sanitary conditions. Social conflicts (e.g. civil wars) impact food production and drain resources from potential disease control programmes, increasing undernourishment and individual’s susceptibility to diseases [37, 38].

Ethiopan malaria scenario may be more influenced by other variables than minimum temperature. The aim of this study is to show that other variables than minimum temperature could be involved in the malaria dynamics in Ethiopia, from 1985 to 2007. Additionally, the dynamics of *Plasmodium vivax* were considered, which were lacking from the [16] study despite of available data.

## Methods

Population ecologists have developed a simple framework, which helps to explore the contributions of endogenous and exogenous processes on population dynamics. Briefly, density-dependent (endogenous process) occurs when the per capita population growth rate (R) is determined by population's previous size. An exogenous process occurs when some variable affects the population but is not affected by the changes it causes. Climate variables and governmental disease control policies are known examples of exogenous pulse/press perturbations with relation to diseases. Both, endogenous and exogenous processes may operate to determine the dynamic behavior of a particular population through time [39–41]. Therefore, a more complete understanding of the dynamics of a population is achieved when both endogenous and exogenous processes are considered. Using this framework, recent studies have captured the trends of measles, tuberculosis, HIV, malaria and pertussis at city, country and global scales [19, 42–45].

The ecological principles mentioned above have analogies in epidemiological processes. Following the introduction of an infected individual in a naïve population, the infected class (*I*_*t*_) is expected to grow exponentially driven by R_0_, the basic reproductive number, since there are almost unlimited susceptible individuals (resources). Nevertheless, as the infected class grows, the susceptible pool is depleted and declines the per infected transmission rate. R_0_ becomes R_E_, the realized reproductive number, a process known as self-limiting, analogous to the principle of intra-specific competition [19, 42, 45]. Higher infected class may increase contact rate enhancing transmission rate, analogous to the intra-specific cooperation principle. The per capita population rate of change (R) is the corner stone of the framework adopted in this study [39–41]. R can be estimated by the natural log differences between actual and past numbers of infected individuals, which are adopted in this work.

The recent study of 2020 [16] suggested that the malaria dynamics in Oramia region (Ethiopia) showed distinct chronological domains, from 1985 to 1998 and from1998 to 2007. Hence, the time series from 1985–1998 and from 1998 to 2007 were re-analysed. In advance, the malaria dynamics showed signs of a negative correlation between *RI*_*t*_ and *I*_*t-1*_ (infected population size class) for both periods and *Plasmodium* spp. (see Table 1), which can be captured by the following Ricker model:

1 $$ R_I = R_{\max} \left( {1 - \left( {\frac{I_{t - 1}}{K}} \right)^{Q} } \right) $$

where *R*_*I*_ is the per capita growth rate of the infected class, *R*_*max*_ is maximum population rate of change (analogous to R_0_), *I*_*t* − 1_ is the infected class (annual averages of malaria cases) in the previous year, K is the stable malaria incidence carrying capacity. *Q* is the pendent when *R*_*I*_ is zero at the carrying capacity of infectious class and measures the degree intraspecific competition, self-limiting, around *K*.

**Table 1:** The logistic model’s results for *P. falciparum* and *P. vivax* from 1985 to 1998 and from 1998 to 2007 without and with the lateral effects of per capita GDP (GDP), as a proxy of poverty, human density (Density), minimum temperature (Temperature), New HIV cases (HIV) and deaths related to civil conflicts (Battle)

Pathogen	Period	Model	Variables		Parameters				R^2^	AIC
					Rmax	K	Q	a		
*P. falciparum*	1985–1998	1			1.82	549.87	0.467		0.499	25.011
*P. falciparum*	1985–1998	2	GDP		1.94	1503.45	0.44	− 4.87	0.501	24.147
*P. falciparum*	1985–1998	3	Density		1.94	856.05	0.44	− 5.934	0.483	24.605
*P. falciparum*	1985–1998	4	HIV		1.94	633.6	0.44	− 0.00016	0.438	24.58
*P. falciparum*	1985–1998	5	Temperature		1.94	125.07	0.44	35.63	0.477	24.824
*P. falciparum*	1985–1998	6	Battle		1.94	601.411	0.44	− 0.0017	0.5766	15.728
*P. falciparum*	1985–1998	7	Temperature	GDP	0.51	1588.33	0.44	− 0.3799	0.65	28.262
*P. falciparum*	1985–1998	8	Temperature	HIV	0.51	92.63	2.27	− 0.00019	0.734	14.662
*P. falciparum*	1985–1998	9	Temperature	Density	0.51	219.912	2.27	0.795	0.638	28.854
*P. falciparum*	1985–1998	10	Temperature	Battle	0.51	751.7	2.27	− 0.00021	0.478	10.865
*P. falciparum*	1985–1998	11	GDP	Density	0.51	519.81	2.27	0.0182	0.624	29.206
*P. falciparum*	1985–1998	12	GDP	HIV	0.51	628.9	2.27	7.12E–06	0.631	29.059
*P. falciparum*	1985–1998	13	GDP	Battle	0.51	751.3	2.27	− 1.2E–05	0.722	10.918
*P. falciparum*	1985–1998	14	HIV	Density	0.51	794.7	2.27	0.000291	0.421	13.449
*P. falciparum*	1985–1998	15	HIV	Battle	0.51	743.5	2.27	− 2.2E–08	0.747	10.763
*P. falciparum*	1985–1998	16	Density	Battle	0.51	751.9	2.27	− 0.00005	0.723	10.859
*P. vivax*	1985–1998	17			3.01	524	0.292		0.352	35.32
*P. vivax*	1985–1998	18	GDP		3.01	2143.56	0.319	− 8.27	0.784	33.971
*P. vivax*	1985–1998	19	Density		3.01	− 498.96	0.319	19.82	0.849	31.683
*P. vivax*	1985–1998	20	HIV		3.01	226.5	0.319	0.00059	0.867	30.662
*P. vivax*	1985–1998	21	Temperature		3.01	− 672.8	0.319	101.5	0.791	35.11
*P. vivax*	1985–1998	22	Battle		3.01	629.06	0.319	− 0.0054	0.507	23.015
*P. vivax*	1985–1998	23	Temperature	GDP	3.01	1784.74	0.319	− 0.546	0.79	34.762
*P. vivax*	1985–1998	24	Temperature	Density	3.01	− 517.495	0.319	1.715	0.845	31.21
*P. vivax*	1985–1998	25	Temperature	HIV	3.01	224.9	0.319	5.05E–05	0.865	30.635
*P. vivax*	1985–1998	26	Temperature	Battle	3.01	627.2	0.319	− 4.3E–05	0.548	23.054
*P. vivax*	1985–1998	27	GDP	Density	3.01	− 249.573	0.319	0.077	0.805	33.489
*P. vivax*	1985–1998	28	GDP	HIV	3.01	225.3	0.319	0.000031	0.868	30.743
*P. vivax*	1985–1998	29	GDP	Battle	3.01	629.6	0.319	− 2.7E–05	0.584	22.943
*P. vivax*	1985–1998	30	HIV	Density	3.01	186.2	0.319	− 0.00022	0.313	14.311
*P. vivax*	1985–1998	31	HIV	Battle	3.01	657.7	0.319	− 3.5E–07	0.343	17.232
*P. vivax*	1985–1998	32	Density	Battle	3.01	616.9	0.319	− 0.00008	0.596	23.321
*P. falciparum*	1998–2007	33			18.01	72.46	0.094		0.369	31.429
*P. falciparum*	1998–2007	34	GDP		0.47	− 614.852	2.061	4.38	0.611	29.549
*P. falciparum*	1998–2007	36	Density		0.47	2710.63	2.061	− 35.26	0.887	19.969
*P. falciparum*	1998–2007	37	HIV		0.47	8708	2.061	− 0.0091	0.942	14.247
*P. falciparum*	1998–2007	38	Temperature		0.47	1743.2	2.061	− 118.7	0.653	28.761
*P. falciparum*	1998–2007	39	Battle		0.47	237.395	2.061	0.0101	0.383	26.384
*P. falciparum*	1998–2007	40	Temperature	GDP	0.47	421.205	2.061	− 0.062	0.575	30.109
*P. falciparum*	1998–2007	41	Temperature	HIV	0.47	− 568.6	2.061	1.328	0.930	15.878
*P. falciparum*	1998–2007	42	Temperature	Density	0.47	2567.31	2.061	− 2641	0.901	19.122
*P. falciparum*	1998–2007	43	Temperature	Battle	0.47	2373	2.061	0.00087	0.383	26.381
*P. falciparum*	1998–2007	44	GDP	HIV	0.47	− 371.2	2.061	0.000062	0.882	20.291
*P. falciparum*	1998–2007	45	GDP	Density	0.47	2023.22	2.061	− 0.125	0.705	27.531
*P. falciparum*	1998–2007	46	GDP	Battle	0.47	237.4	2.061	0.000052	0.383	26.384
*P. falciparum*	1998–2007	47	HIV	Density	0.47	− 580.3	2.061	0.00023	0.941	15.345
*P. falciparum*	1998–2007	48	HIV	Battle	0.47	237.3	2.061	1.7E – 06	0.382	26.372
*P. falciparum*	1998–2007	49	Density	Battle	0.47	237.4	2.061	0.000152	0.384	26.388
*P. vivax*	1998–2007	50			0.5	185	0.094		0.401	20.519
*P. vivax*	1998–2007	51	GDP		0.5	1833.35	0.784	− 7.306	0.661	17.219
*P. vivax*	1998–2007	52	HIV		0.5	− 357.1	0.784	0.0016	0.789	16.364
*P. vivax*	1998–2007	53	Density		0.5	1379.7	0.784	− 15.47	0.527	18.912
*P. vivax*	1998–2007	54	Temperature		0.5	2822.9	0.784	− 199.8	0.602	17.76
*P. vivax*	1998–2007	55	Battle		0.5	203.054	0.784	0.247	0.406	13.341
*P. vivax*	1998–2007	56	Temperature	GDP	0.5	2627.7	0.784	− 0.908	0.822	13.245
*P. vivax*	1998–2007	57	Temperature	Density	0.5	1535.2	0.784	− 1.451	0.590	18.046
*P. vivax*	1998–2007	58	Temperature	HIV	0.5	6059	0.784	− 0.0005	0.757	14.159
*P. vivax*	1998–2007	59	Temperature	Battle	0.5	203.429	0.784	0.019	0.407	13.341
*P. vivax*	1998–2007	60	GDP	Density	0.5	1544.97	0.784	− 0.089	0.660	17.324
*P. vivax*	1998–2007	61	GDP	HIV	0.5	1455	0.784	− 6E–06	0.707	16.831
*P. vivax*	1998–2007	62	GDP	Battle	0.5	202.4	0.784	0.00129	0.406	13.368
*P. vivax*	1998–2007	63	HIV	Density	0.5	− 452.9	0.784	0.00217	0.837	12.56
*P. vivax*	1998–2007	64	HIV	Battle	0.5	205.9	0.784	3.5E–0 6	0.407	13.283
*P. vivax*	1998–2007	65	Density	Battle	0.5	201.2	0.784	0.0041	0.407	13.323

The results suggest a weak effect of minimum temperature to malaria dynamics for all periods and species. Models were compared and selected for each period and for each species based on high R^2^ and lower AIC. From 1985 to 1998 for *P. falciparum*, the models 11, 13, 16 delivered the best results. For the same period and for *P. vivax* the models 25 and 28 delivered the best results. From 1998 to 2007 (*P. falciparum*) the models 37, 41 and 47 delivered the best results. From 1998 to 2007 (*P. vivax*) the models 56 and 63 delivered the best results. The logistic model without exogenous effects can be interpreted as a null model and models with exogenous effects as alternative models

The effect of exogenous variables on malaria dynamics will be assessed using Royama methodology [39], whom classified three basic exogenous effects on the logistic model (Eq. 1): vertical, lateral and non-linear effects. A vertical effect on R changes the relative position of the R function by changing proportionally both *Rmax* (y-scale) and *K* (x-scale) intercepts but not he non-linear parameter *Q*. Lateral effects occur when only the *K* is affected (Eq. 2), and the non-linear effects when the variable affects both *Rmax* and *K*, but disproportionally. This study uses inter-annual data instead of monthly data, mainly because there are no monthly data for human population size, nor HIV indices among other variables. This leaves us with less degree of freedom (annual data), thus the study explores the lateral effects of the variables. The lateral exogenous effects can be tested as follows [39–41]:

2 $${R_I} = {R_{\max }}\left( {1 - \left( {\frac{{{I_{t - 1}}}}{{K + {a^*}{V_t}}}} \right){\mathbin{\lower.3ex\hbox{$\buildrel\textstyle\rightarrow\over
{\smash{\leftarrow}\vphantom{_{\vbox to.5ex{\vss}}}}$}} ^Q}} \right)$$

where *a* is the linear coefficient that measures the effect of any exogenous variable (*V*_*t*_) on the self-limiting model parameters. Other parameters are as above.

The HIV national new cases per year, population density and death related to civil conflicts, as a proxy of social instability (Battle in Table 1), were obtained from the World Bank (https://www.worldbank.org/en/home) and World Health Organization (https://www.who.int/) websites. The national trends were interpreted as a proxy for what occurs at a smaller scale (Oromia region) [19, 45]. Falciparum and vivax malaria cases and the minimum Temperature (°C) data was obtained from the study [16], where only the minimum Temperature (°C) was considered. Equation 1 measures the endogenous contribution to malaria dynamics. Equation 2 measures the effects of the mentioned exogenous variables. This study presents the results of the effects of each of the variables, but,mainly focusses on the interaction between the exogenous variables.

The best model was selected based on the R^2^ (Coefficient of determination) and on the Akaike information criteria (AIC) [46] for each species and period. The predictability, the goodness of fit (R^2^) of a model usually increase by adding more variables and AIC penalizes the model in regard to the number of variables included, meaning that less variables needed to explain an observed pattern more parsimonious is the model. Equations 1 and 2 were fitted using the nls library in R through non-linear regression analysis [47].

## Results

The malaria increase in numbers could be captured by the logistic growth model (Table 1, model 1, 17, 33 and 50) for the distinct species and periods. The logistic model alone explained more than 40% of the variance of the R levels, the self-limiting process between the infectious class and susceptible pool. Table 1 also shows the effects of the mentioned variables on carrying capacity (K) of the infectious class.

The logistic models with the interaction between GDP and human population size (model 11), GDP and social instability (model 13). HIV new cases and social instability (Battle in Table 1, model 14) and human population size and social instability (model 16) delivered similar and the bests results (higher R^2^ and lower AIC) for *P. falciparum,* from 1985 to 1998.

For *P. vivax*, the interaction between the effects of HIV new cases and GDP (model 28) delivered the best result (Table 1. Higher R^2^ and lower AIC). The interaction between the effects of the minimum temperature with HIV new cases (model 25) gave similar results, from 1985 to 1998.

From 1998 to 2007, the model for *P. falciparum* with HIV new cases as exogenous lateral effect without interactions delivered the best results (model 37) followed by the modes with interactions between HIV new cases and population size (model 47) and minimum temperature and HIV new cases (model 41), respectively. For *P. vivax*, the interaction between HIV new cases and human density (model 63) gave the best model (Higher R^2^ and lower AIC values) followed by the interaction between minimum temperature and GDP (model 56, Table 1).

## Discussion

The results suggest that the interactions between HIV new cases, human density and GDP gave better results than models without exogenous variables and models with exogenous variables, but without interactions suggesting that climate change had an almost negligeable effect in terms of *P. falciparum* and *P. vivax* dynamics in Ethiopia. In this study, the manner in which the effects of these variables could interact to explain the changes in malaria dynamics will be discussed.

Both malaria and HIV are related to poverty [24–27], which can be exacerbated by civil conflicts [37, 38]. Poverty may force individuals to engage in risk behaviours (to avoid extreme poverty) and be more susceptible to contract HIV (sex workers), and hence, malaria [19–21].The spread of HIV could remove the herd immunity effect. The interaction between malaria and HIV is suspected to be synergetic [20], because malaria-infected individuals show an increase of the HIV host cells in the immunological system. This could produce a longer period of the acute phase of HIV, increasing infectivity between individuals and susceptibility to future malaria infections. Besides the synergy, the effects of HIV seem to be greater than the effects of malaria on the spread of HIV, because the infection period and AIDS stage may last for years reducing the immune defense efficiency [21–24]. The spread of HIV could expand the population at risk of contracting malaria in the growing population of Ethiopia, which may increase the contact rate and create mosquitoes breeding sites trough deforestation. Civil conflicts affect crops, favouring undernourishment (susceptibility), reducing health care investments and intensify human movement (refugees), which can amplify malaria distribution and burden [48, 49].

All these variables may also explain the malaria decline period and serve as alternative hypothesis to the claimed effect of minimum temperature. From 1998 to 2007, GDP levels increased, HIV levels increased but at lower rates (almost reaching an equilibrium), civil conflicts diminished and population density rose, but, as for HIV, at lower rates. GDP levels started to increase since 1990 and accelerated at the end of time series (1998 to 2007). Higher income may allow individuals to invest out-of-pocket for treating malaria. From 1998 to 2000, social instability peaked and dropped at low levels from 2000 onwards. The increase in income, the reduction of civil instability, the increase of HIV and human population size at low rates, could interact to explain the malaria decrease burden from 1998 to 2007.

It is important not to rule out climate change. Minimum temperature also delivered good results as exogenous force on the logistic model, but only when other variables were added (the models with interactions). This could be simply because of the stronger effects of others variables, and/or the fact that interannual data was used instead of monthly data, which could capture more precisely the malaria cases peaks. Climate variables could always provide an initial set of potential variables, which could influence the dynamics of malaria (not only minimum temperature). Temperature can accelerate larval development rate (including more life-cycles per season) and reduce the differences between seasons. Local rises in temperature could interact with increase in human density and HIV to explain the increase of malaria cases during the first period (1985–1998).

A recent study revised the climate effect on *P. falciparum* in Kericho tea plantations [19] and the results from this study showed that temperature (maximum, mean and minimum) and rain (average) had an almost negligeable role on malaria dynamics. The interaction of human population size and HIV levels had more contribution in explaining malaria dynamics than climate change, which reinforces the results from this study for Ethiopia, where others variables could have more influence on malaria burden than climate change [19]. In 2000, several countries joined in a global effort to halt and begin to reverse the incidence of malaria by 2015 (Millennium Development Goals 6- Target 6C) [26, 27]. African countries received international funding and developed efficient national strategies to achieve the Millennium Development Goals [50–54]. In many African countries, this occurred in 2005, one year after USA made a massive investment to increase ART (Antiretroviral Therapy) coverage in sub-Saharan Africa [27, 28].

In Ethiopia, the interaction between the effects of human density, HIV, GDP, and in some cases civil disorder could explain the decline of cases for *P. falciparum* (and similarly for *P. vivax*) from 1998 to 2005 in Ethiopia. This study also highlights that efforts to control malaria and HIV could interact in order to achieve the malaria Millennium Development Goals 6- Target 6C [54–58].

## Conclusion

Here, the Population Ecology Theory was adopted to re-explore malaria cases dynamics in Ethiopia. This study presents a distinct scenario to explain that others large-scale phenomena (HIV, population size, among others) could have influenced malaria dynamics at a higher level than climate change.

The framework employed is based on the per capita population rate of change (*R*_*I*_), which is surrounded by plausible ecological principles and is hence an advantageous starting point to explore disease dynamics. Any government may disentangle *R*_*I*_ in its components (new per capita infections and per capita mortality), explore which of them are most important for *R*_*I*_ trends and explore the contributions of endogenous and exogenous processes. Hence, this approach, based on simple principles based on population ecology theory, could be included as a supplement to WHO reports with minimal cost- and time-demanding efforts, which could provide insights and hypotheses and may facilitate the testing and estimation of the drivers of disease dynamics.
